# Resolvin D1 and D2 Inhibit Transient Receptor Potential Vanilloid 1 and Ankyrin 1 Ion Channel Activation on Sensory Neurons via Lipid Raft Modification

**DOI:** 10.3390/ijms21145019

**Published:** 2020-07-16

**Authors:** Maja Payrits, Ádám Horváth, Tünde Biró-Sütő, János Erostyák, Géza Makkai, Éva Sághy, Krisztina Pohóczky, Angéla Kecskés, Miklós Kecskés, János Szolcsányi, Zsuzsanna Helyes, Éva Szőke

**Affiliations:** 1 Department of Pharmacology and Pharmacotherapy, Medical School, University of Pécs, Szigeti str. 12, H-7624 Pécs, Hungary; payrits.maja@gmail.com (M.P.); tunde.suto@aok.pte.hu (T.B.-S.); saghy.eva@med.semmelweis-univ.hu (É.S.); pohoczkykriszti@gmail.com (K.P.); angela.kecskes@aok.pte.hu (A.K.); janos.szolcsanyi@aok.pte.hu (J.S.); zsuzsanna.helyes@aok.pte.hu (Z.H.); eva.szoke@aok.pte.hu (É.S.); 2 János Szentágothai Research Centre and Centre for Neuroscience, University of Pécs, Ifjúság str. 20, H-7624 Pécs, Hungary; erostyak@fizika.ttk.pte.hu (J.E.); gezamakkai@gmail.com (G.M.); kecskes.miklos@pte.hu (M.K.); 3 Department of Experimental Physics, Faculty of Sciences, University of Pécs, Ifjúság str. 6, H-7624 Pécs, Hungary; 4 Department of Pharmacology and Pharmacotherapy, Semmelweis University, Nagyvárad sq. 4, H-1089 Budapest, Hungary; 5 Department of Pharmacology, Faculty of Pharmacy, University of Pécs, Szigeti str. 12, H-7624 Pécs, Hungary; 6 Institute of Physiology, Medical School, University of Pécs, Szigeti str. 12, H-7624 Pécs, Hungary

**Keywords:** Resolvin D1, Resolvin D2, lipid rafts, transient receptor potential channel, sensory neuron, nerve terminal

## Abstract

Transient Receptor Potential Vanilloid 1 and Ankyrin 1 (TRPV1, TRPA1) cation channels are expressed in nociceptive primary sensory neurons and regulate nociceptor and inflammatory functions. Resolvins are endogenous lipid mediators. Resolvin D1 (RvD1) is described as a selective inhibitor of TRPA1-related postoperative and inflammatory pain in mice acting on the G protein-coupled receptor DRV1/GPR32. Resolvin D2 (RvD2) is a very potent TRPV1 and TRPA1 inhibitor in DRG neurons, and decreases inflammatory pain in mice acting on the GPR18 receptor, via TRPV1/TRPA1-independent mechanisms. We provided evidence that resolvins inhibited neuropeptide release from the stimulated sensory nerve terminals by TRPV1 and TRPA1 activators capsaicin (CAPS) and allyl-isothiocyanate (AITC), respectively. We showed that RvD1 and RvD2 in nanomolar concentrations significantly decreased TRPV1 and TRPA1 activation on sensory neurons by fluorescent calcium imaging and inhibited the CAPS- and AITC-evoked ^45^Ca-uptake on TRPV1- and TRPA1-expressing CHO cells. Since CHO cells are unlikely to express resolvin receptors, resolvins are suggested to inhibit channel opening through surrounding lipid raft disruption. Here, we proved the ability of resolvins to alter the membrane polarity related to cholesterol composition by fluorescence spectroscopy. It is concluded that targeting lipid raft integrity can open novel peripheral analgesic opportunities by decreasing the activation of nociceptors.

## 1. Introduction

The Transient Receptor Potential (TRP) Vanilloid 1 (TRPV1) and Ankyrin 1 (TRPA1) cation channels are co-localized on capsaicin (CAPS)-sensitive polymodal nociceptors, and mediate pain and neurogenic inflammation [1,2,3,4]. TRPV1 is gated by chemical irritants, such as CAPS, resiniferatoxin, several endogenous arachidonic- or other fatty-acid metabolites, protons (pH < 6.0) and noxious heat (>43 °C) [5,6,7,8,9,10]. TRPA1 is gated by a variety of exogenous (such as allyl-isothiocyanate—AITC) and endogenous ligands (like primary amine metabolites, methylglyoxal [11,12,13]), cold (<17 °C), and mechanical stimuli [14,15,16]. Pro-inflammatory neuropeptides, such as calcitonin gene-related peptide (CGRP) and substance P are released from the activated TRPV1 and TRPA1-expressing sensory nerves and induce neurogenic inflammation (vasodilatation, plasma protein extravasation, and inflammatory cell stimulation) in the innervated area [17,18,19]. Neurogenic inflammation is an important pathophysiological component of diseases like asthma, rheumatoid arthritis, psoriasis, and inflammatory bowel diseases, and cannot be inhibited by any currently available drugs [20,21,22]. Furthermore, chronic neuropathic pain, in which the CAPS-sensitive nerves play an important role, is also an unmet medical need. Therefore, inhibiting function of TRPV1 and TRPA1-expressing nociceptors is the focus of novel pharmacological approaches. Great effort has been dedicated to developing small-molecule TRPV1 and TRPA1 antagonists [2,3,23,24,25,26,27,28], but disrupting lipid rafts surrounding these TRP channels could also be a promising alternative way to inhibit receptor activation.

Lipid rafts are microdomains rich in cholesterol, sphingomyelins (SM), and gangliosides [29]. Cholesterol depletion by methyl β-cyclodextrin incubation resulted in impaired CAPS-evoked currents in dorsal root ganglion (DRG) neurons [30]. We proved that pharmacological depletion of SMs, cholesterol or gangliosides diminished TRPV1 and TRPA1 channel activation both on transfected cells and native sensory neurons [31,32]. A very recent study showed that lipid raft destabilization by methyl β-cyclodextrin also inhibits the responses of mouse TRPA1 to bacterial lipopolysaccharides and cold [33]. TRPA1 was described as being located in cholesterol-rich domains. The authors identified by in silico investigation cholesterol recognition amino acid consensus motifs in the TM2 and TM4 segments, which had a role in the inhibition of chemical activation of mouse TRPA1 by cholesterol depletors [34]. Cholesterol might influence TRP channel function by direct interaction with ion channels [34,35,36]. Sphingomyelinase hydrolyzes SM to phosphocholine and ceramide [37,38]. It did not only act on the sensory neuronal cell bodies, but also on the nerve terminals diminishing TRPV1 and TRPA1 activation-induced CGRP release, as a good indicator of channel inhibition [31]. The described changes of the plasma membrane composition related to lipid raft disruption could be investigated in a reliable manner by fluorescence spectroscopy, as have been reported earlier [31,32,39,40].

**Figure 1:**
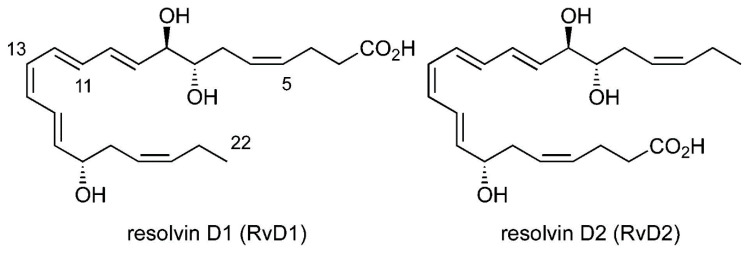
Structure of RvD1 and RvD2.

Identifying the endogenous inhibitors of TRPV1 and TRPA1 channels could open novel perspectives to understand their pharmacological opportunities. Resolvins are lipid mediators with pro-resolving and anti-inflammatory functions [41,42]. Resolvin D1 (RvD1) is derived from docosahexaenoic acid (Figure 1) [42] and is a potent inhibitor of inflammatory and postoperative pain, but its molecular mechanism of action is unclear [43,44,45]. RvD1 was described as a selective inhibitor of TRPA1 in DRG neurons [43]. Resolvin D2 (RvD2)—which is also a docosahexaenoic acid derivate (Figure 1)—is a regulator of leukocyte functions and controls sepsis [46]. RvD2 has been reported as a very potent TRPV1 and TRPA1 inhibitor in DRG neurons, which inhibited cation influx in nanomolar concentration (IC_50_ 0.1 nM for TRPV1 and IC_50_ 2.1 nM for TRPA1) [47]. Peripheral and central administration of its low dose (0.01–10 ng) decreased acute, subacute, and persistent inflammatory pain in mouse models via TRPV1/TRPA1-independent mechanisms. This effect was suggested to be mediated by its specific G protein-coupled receptor (GPR) [47], which was later identified by direct binding of ^3^H-labeled RvD2. It is called GPR18 when described on human leukocytes, including polymorphonuclear neutrophil cells, monocytes, and macrophages [48]. DRV1/GPR32 is the receptor for RvD1 identified in human leukocytes [49,50].

The present study investigated the effects of RvD1 and RvD2 on TRPV1 and TRPA1 receptor activation on trigeminal sensory neurons and peripheral sensory nerve endings and the involvement of lipid raft modification around these nocisensor ion channels in their mechanisms of action.

## 2. Results

### 2.1. RvD1 and RvD2 Inhibit TRPA1 and TRPV1 Receptor Activation-Mediated Ca^2+^-Influx in Cultured Trigeminal Ganglion (TG) Neurons

**Figure 2:**
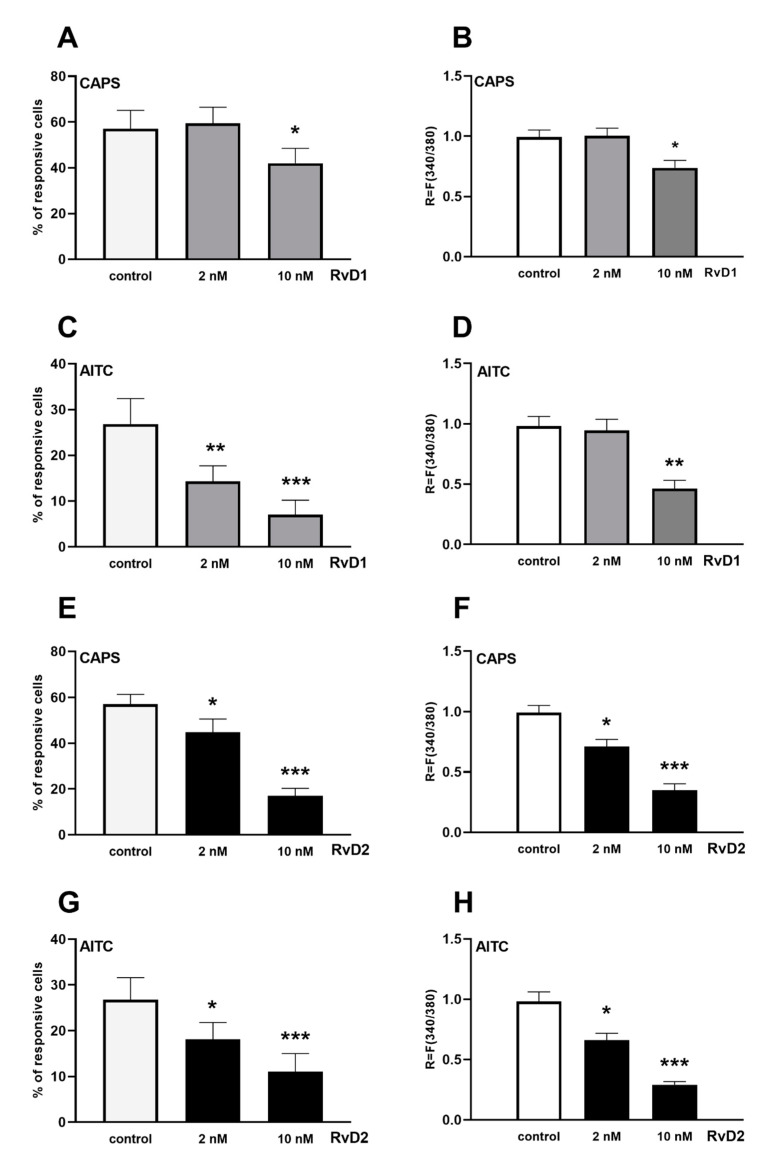
Effect of RvD1 and RvD2 on TRPV1 and TRPA1 receptor activation on cultured trigeminal ganglion sensory neurons. (**A**,**E**): The percentage of responsive cells to capsaicin (CAPS) is presented after (**A**): RvD1 (2 and 10 nM, *n* = 94–106 cells per group) and (**E**): RvD2 (2 and 10 nM, *n* = 102–108 cells per group) administration. (**B**,**F**): Change in the fluorescence ratio (*R* = *F*340/*F*380) after TRPV1 receptor activation in CAPS-sensitive cells is presented after (**B**): RvD1 and (**E**): RvD2 treatment. (**C**,**G**): The percentage of responsive cells to allyl-isothiocyanate (AITC) is presented after (**C**): RvD1 (2 and 10 nM, *n* = 84–170 cells per group) and (**G**): RvD2 (2 and 10 nM, *n* = 89–170 cells per group) administration. (**D**,**H**): Change in the fluorescence ratio (*R* = *F*340/*F*380) after TRPA1 receptor activation in AITC-sensitive cells is presented after (**D**): RvD1 and (**H**): RvD2 treatment. Ca^2+^-responses are presented in % of total number of examined neurons. (* *p* < 0.05, ** *p* < 0.01, *** *p* < 0.001 (control vs. treated, one-way ANOVA, Dunnett’s post hoc test).

Firstly, the percentage of responding neurons with Ca^2+^-influx to 330 nM CAPS and 200 μM AITC was determined in control plates. In control plates, CAPS- and AITC-induced Ca^2+^-influx were detected in 57.1 ± 8% (60 out of 104, the *R* value was 0.99 ± 0.05) and 25.6 ± 5.6% (43 out of 170, the *R* value was 0.98 ± 0.07) of the cells, respectively. RvD1 treatment in 10 nM significantly decreased not just the proportion of cells responding to CAPS and AITC resulting in 42.1 ± 6.5% (40 out of 94) and 7 ± 3.2% (8 out of 91) responsive cells, respectively, but also the *R* values, resulting in *R* = 0.73 ± 0.07 and 0.46 ± 0.06, respectively. After 2 nM RvD1 incubation, the percent of responsive cells to CAPS was unaffected (59.5 ± 7% (63 out of 106)), while it diminished the AITC-evoked responses to 14.3 ± 3.4% (12 out of 84) (Figure 2A–D).

**Figure 3:**
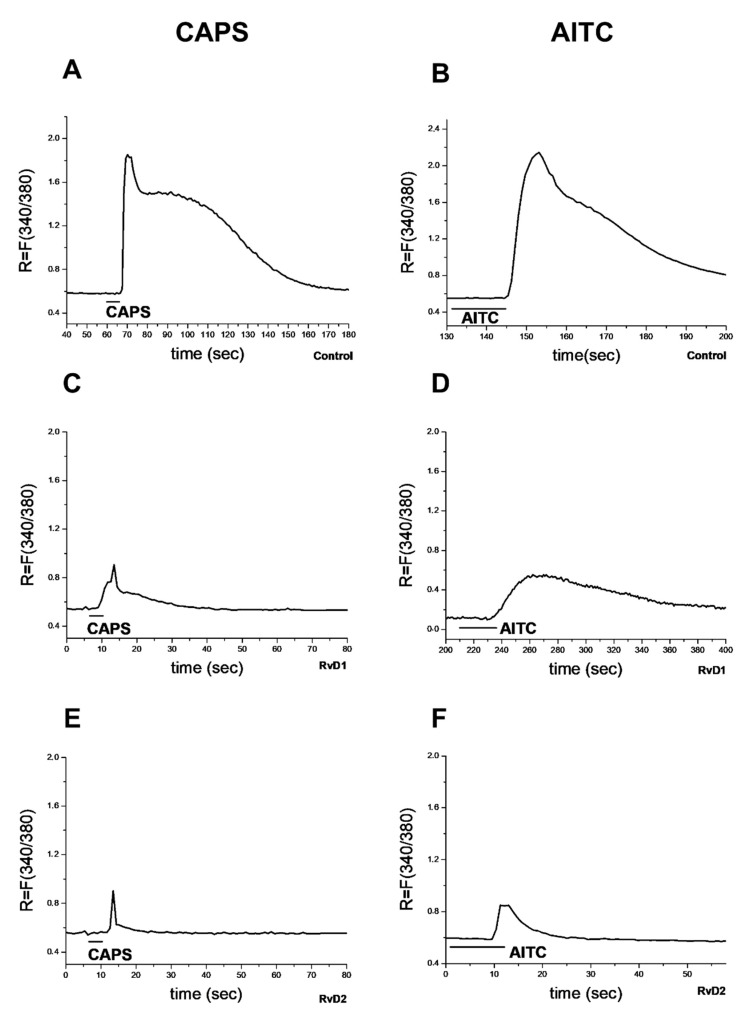
Effect of RvD1 and RvD2 on TRPV1 and TRPA1 receptor activation on cultured TG sensory neurons. Increases in *R* = 340/380 fluorescence in fura-2 loaded cultured TG neurons. Original recording from CAPS (**A**,**C**,**E**)- and AITC (**B**,**D**,**F**)-sensitive neurons on control (**A**,**B**), RvD1-treated (**C**,**D**) or RvD2-treated (**E**,**F**) plates.

Significant decreases in the percent of CAPS- and AITC-sensitive cells were observed after 2 and 10 nM RvD2 incubation, resulting in 44.8 ± 5.8% (48 out of 108) and 17 ± 3.3% (18 out of 102) in the case of CAPS and 18.1 ± 3.7% (20 out of 108) and 11 ± 4.1% (9 out of 89) responsive cells in the case of AITC, respectively (Figure 2E–H). The related *R* values in the case of CAPS have been decreased also to 0.71 ± 0.05 and 0.35 ± 0.05 after 2 and 10 nM RvD2 incubation, respectively, and similar decreases were observed in the case of AITC, resulting in 0.66 ± 0.05 and 0.29 ± 0.02 *R* values, respectively (Figure 2E–H). Original recordings of CAPS- and AITC-induced Ca^2+^-influx in TG neurons are presented in Figure 3.

**Figure 4:**
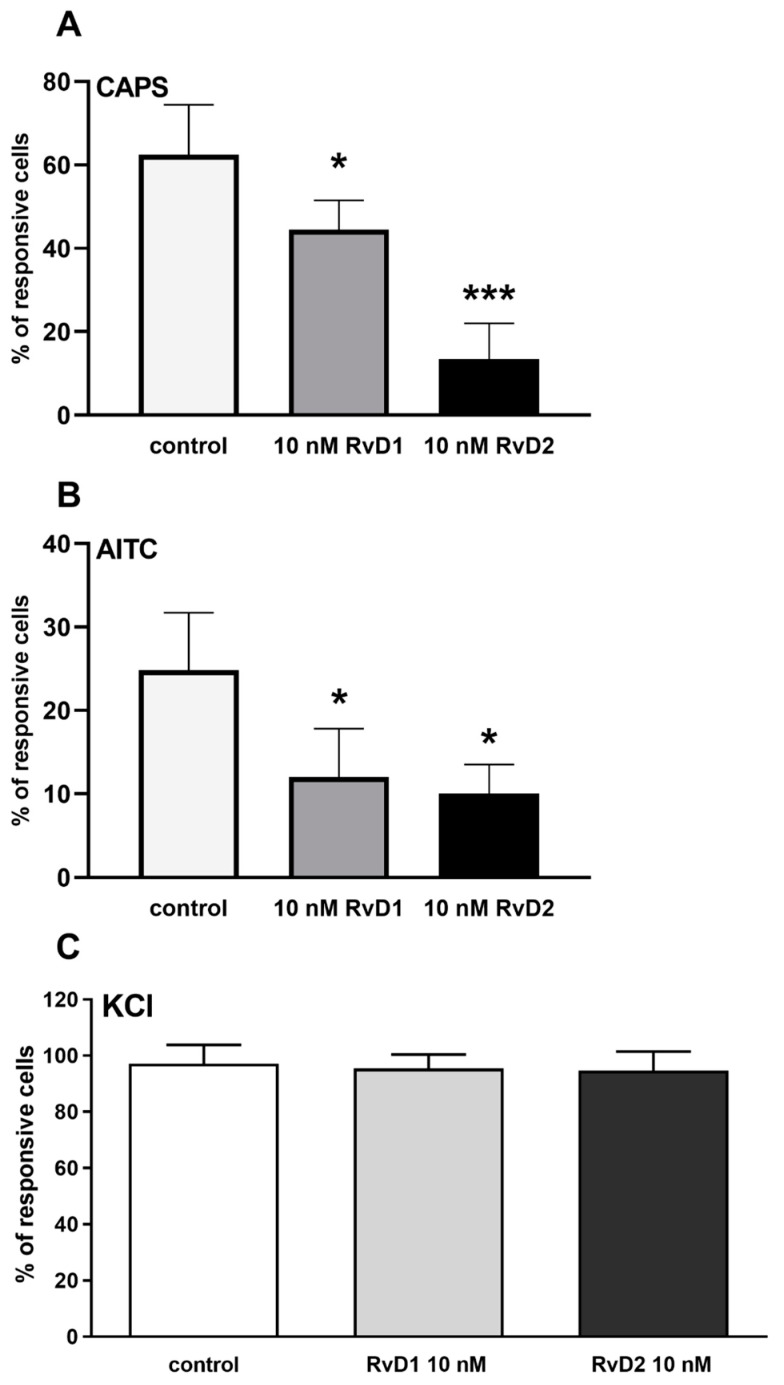
Effect of RvD1 and RvD2 on TRPV1 and TRPA1 receptor activation on cultured TG sensory neurons after PTX treatment and on KCl-evoked voltage gated Ca^2+^ channel activation. (**A**): The percentage of responsive cells to CAPS is presented after RvD1 or RvD2 (both of them 10 nM) administration. (**B**): The percentage of responsive cells to AITC is presented after RvD1 or RvD2 (both of them 10 nM) administration. (**C**): The percentage of responsive cells to KCl is presented after RvD1 or RvD2 (both of them 10 nM) administration. (* *p* < 0.05, *** *p* < 0.001 (control vs. treated, one-way ANOVA, Dunnett’s post hoc test).

The overnight pertussis toxin (PTX) treatment did not prevent the inhibitory effect of the higher doses of RvD1 and RvD2 on TRPV1 and TRPA1 receptor activation (Figure 4A,B). Neither RvD1 nor RvD2 affected the 50 mM KCl-evoked voltage-gated Ca^2+^ channel activation (Figure 4C).

### 2.2. RvD1 and RvD2 Decrease TRPV1 and TRPA1 Activation-Evoked CGRP Release from Peripheral Sensory Nerves

**Figure 5:**
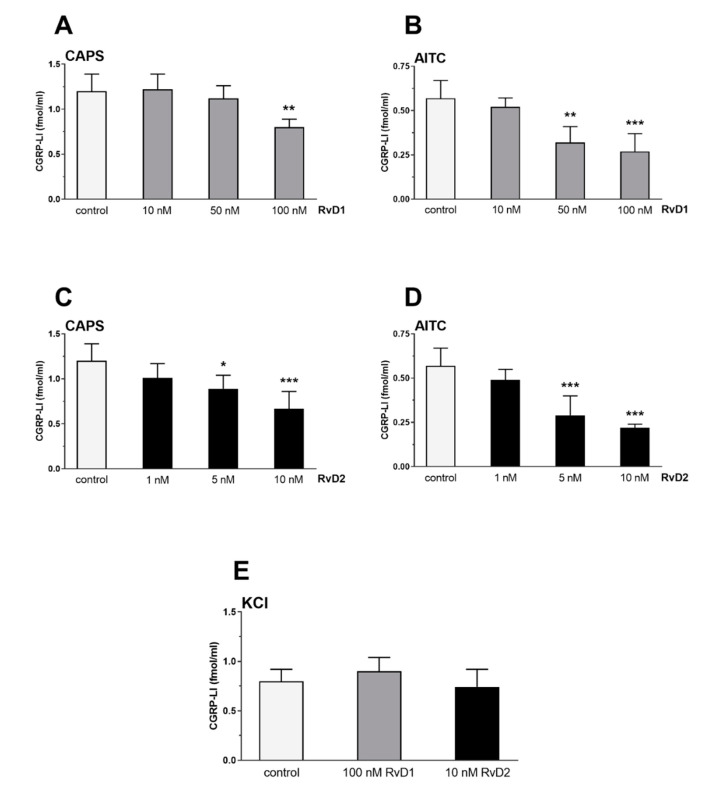
Effect of RvD1 and RvD2 on TRPV1 and TRPA1 receptor activation on peripheral nerve terminals. (**A**,**B**): Effect of 10, 50, and 100 nM RvD1 on 100 nM CAPS-evoked (**A**) or 100 μM AITC-evoked (**B**) CGRP release. (**C**,**D**): Effect of 1, 5, and 10 nM RvD2 on 100 nM CAPS-evoked (**C**) or 100 μM AITC-evoked (**D**) CGRP release. (**E**): Effect of 100 nM RvD1 and 10 nM RvD2 on 50 mM KCl-evoked CGRP release. Each column represents the mean + SEM of values of 6–8 experiments. As this method is a radioimmunoassay, CGRP release is represented as CGRP-like immunoreactivity (CGRP-LI) (* *p* < 0.05, ** *p* < 0.01, *** *p* < 0.001 control vs. treated, one-way ANOVA, Dunnett’s post hoc test).

TRPV1 activation by 100 nM CAPS induced 1.2 ± 0.19 fmol/mg CGRP release from the sensory nerves of the rat trachea. TRPA1 activation by 100 μM AITC evoked 0.57 ± 0.1 fmol/mg CGRP outflow, which is approximately half of the effect of CAPS. RvD1 significantly decreased the CAPS-evoked CGRP release in 100 nM concentration to 0.8 ± 0.09 fmol/mg (Figure 5A) and the AITC-induced peptide outflow in 50 and 100 nM concentration to 0.32 ± 0.09 fmol/mg and 0.27 ± 0.1 fmol/mg (Figure 5B). RvD2 was able to diminish the CGRP release in a ten-fold lower concentration. It decreased significantly the CAPS- and AITC-evoked CGRP release in 5 nM concentration to 0.89 ± 0.15 and 0.29 ± 0.11 fmol/mg, respectively, and in 10 nM concentration to 0.67 ± 0.19 and 0.22 ± 0.02 fmol/mg, respectively (Figure 5C,D). RvD1 and RvD2 did not influence the basal, non-stimulated peptide outflow (data not shown) and KCl-evoked CGRP release (Figure 5E).

### 2.3. RvD1 and RvD2 Inhibit the CAPS- and AITC-Evoked ^45^Ca-Uptake on Chinese Hamster Ovary (CHO) Cells Expressing the Cloned TRPV1 and TRPA1 Receptor

**Figure 6:**
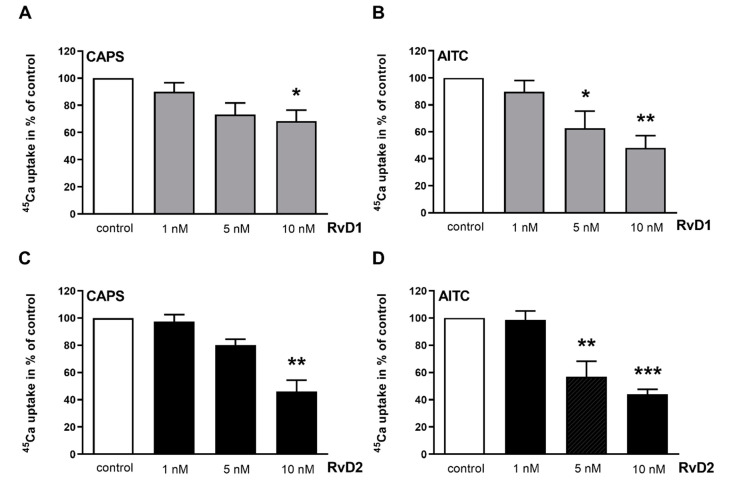
Effect of RvD1 and RvD2 on CAPS- and AITC-evoked ^45^Ca-uptake on CHO cells expressing the cloned TRPV1 and TRPA1 receptor. (**A**,**B**): Effect of RvD1 on TRPV1-expressing (**A**) and TRPA1-expressing (**B**) CHO cells in radioactive ^45^Ca-uptake experiments after CAPS (100 nM) and AITC (100 µM) administration. (**C**,**D**): Effect of RvD2 on TRPV1-expressing (**C**) and TRPA1-expressing (**D**) CHO cells in radioactive ^45^Ca-uptake experiments after CAPS (100 nM) and AITC (100 µM) administration. ^45^Ca-accumulations are presented in % of control (non-treated). Each column represents the mean + SEM of values of nine experiments. (* *p* < 0.05, ** *p* < 0.01, *** *p* < 0.001 (control vs. treated, one-way ANOVA, Dunnett’s post hoc test).

A significant decrease in CAPS-evoked ^45^Ca-accumulation was observed when CAPS was co-administered with 10 nM RvD1 on TRPV1-expressing CHO cells. The percent value of ^45^Ca-uptake relative to the untreated control (100%) was 68.33 ± 18.14% after RvD1 treatment (Figure 6A). The highest concentration of RvD2 was able decreased the CAPS-evoked ^45^Ca-uptake, and the percent value was 46 ± 14.4% relative to the untreated control (Figure 6C).

Both 5 and 10 nM RvD1 and RvD2 significantly diminished the AITC-induced ^45^Ca-uptake relative to the control, and these values were 62.66 ± 21.2% and 48 ± 14.9% in the case of RvD1 and 57 ± 19% and 44 ± 6.2% in the case of RvD2, respectively (Figure 6B,D).

### 2.4. RvD1 and RvD2 Change the Membrane Polarity on CHO Cells

To investigate the effect of RvD1 and RvD2 on lipid raft integrity, fluorescent spectroscopy measurements with 6-dodecanoyl-N,N-dimethyl-2-naphthylamine (Laurdan)—a membrane polarity selective probe—were performed on CHO cells.

**Figure 7:**
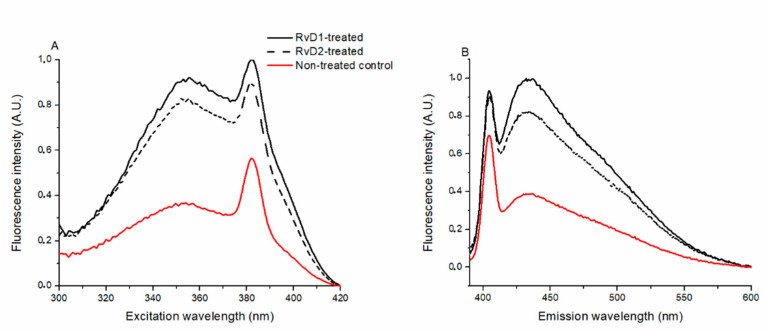
Effect of RvD1 and RvD2 on Laurdan excitation (**A**) and emission (**B**) spectra on CHO cells. Excitation wavelength: 440 nm, emission wavelength: 355 nm. Solid (black) or dashed and solid (red) curves represent the RvD1 or RvD2 and non-treated control samples, respectively.

There is no spectral shift, broadening or any change in the shape of the spectra, but serious intensity change can be seen, which means that the micro-environment of Laurdan is changed by both RvD1 and RvD2 (Figure 7A,B).

**Figure 8:**
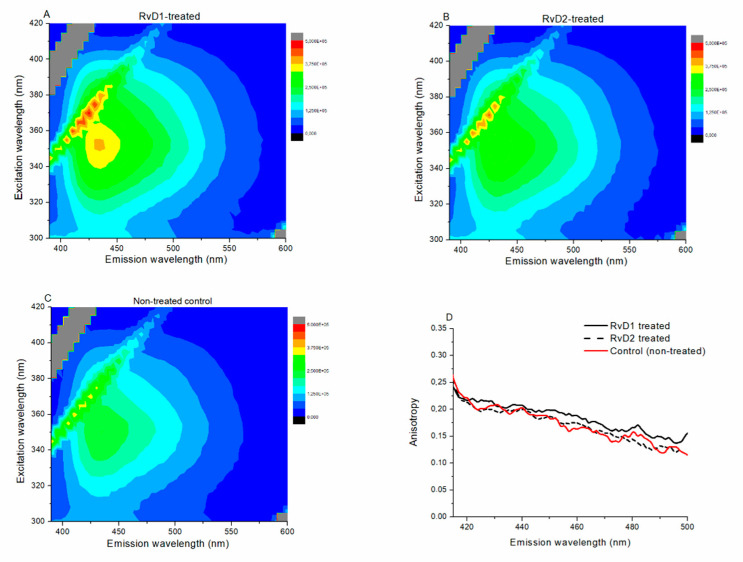
Excitation–emission matrices of RvD1-treated (**A**), RvD2-treated (**B**), and non-treated control (**C**) samples. (**D**): Emission anisotropy spectra of RvD1 (solid black), RvD2 (dashed black), and non-treated control (solid red) samples.

Excitation–emission matrices also show that the RvD1- and RvD2-treated samples (cultured CHO cells) (Figure 8A,B) have higher fluorescence intensity than the non-treated control sample (Figure 8C) on the entire spectral region of fluorescence of Laurdan. The anisotropy values are practically the same on the whole spectral range in the case of RvD1-, RvD2-treated, and non-treated control samples (Figure 8D).

## 3. Discussion

This is the first paper providing evidence that resolvins inhibit the activation of TRPV1 and TRPA1 ion channels on sensory nerve terminals besides the cell bodies via modifying the membrane composition and lipid rafts. Our results on TG neurons confirm that RvD2 is a potent inhibitor of the TRPV1 and TRPA1 receptors in nanomolar concentrations. In contrast to the earlier studies conducted on DRG neurons [47], we showed that RvD1 treatment in 10 nM concentration significantly decreased both the magnitude of Ca^2+^-influx in TG neurons and the proportion of cells responding to TRPV1 activation in addition to TRPA1 stimulation. Similarly to the literature data [47], RvD2 had more pronounced inhibitory action on both TRPV1 and TRPA1 channels. PTX pretreatment did not affect the inhibitory effect of the 10 nM doses of RvD1 and RvD2 on TRPV1 and TRPA1 ion channel activity in cultured TG neurons. Previous studies described that pretreatment of DRG cultures with PTX blocked inhibitory effects of lower concentration (1 nM) of RvE1 on capsaicin-induced TRPV1 activation [51]. Another study described that PTX treatment also blocked the effect of nanomolar doses of RvD1 and RvD2 on TRPV1 and TRPA1 channel activation [47]. Our results with higher doses of resolvins suggests another G-protein-independent way of inhibition. KCl-evoked voltage-gated Ca^2+^ channel activation remained unaltered after RvD1 or RvD2 treatment. The trachea is a good model system to examine the activation of sensory nerve terminals because the nerves are close to the surface and they can be easily stimulated [52,53]. RvD2 concentrations (5 and 10 nM) to reduce TRPV1 and TRPA1 activation on the sensory nerve terminals shown by the decreased CGRP release were similar to that needed for the inhibitory action on the isolated cell bodies. However, 10-fold higher concentrations of RvD1 had to be used to exert similar inhibitory actions to RvD2 on the nerve endings, which might be explained by its weaker penetration abilities into deeper tissue layers. Neither RvD1 nor RvD2 decreased the 50 mM KCl-induced CGRP release.

RvD1 was originally characterized as a selective TRPA1 inhibitor in DRG neurons and human embryonic kidney cells in nanomolar concentration by patch clamp and calcium imaging studies [43,47]. However, RvD1 inhibited fluorescent Ca^2+^-influx into TG sensory neurons in our experimental system in response to both TRPV1 and TRPA1 activations, although higher concentration (10 nM) was needed for TRPV1 inhibition. In contrast to RvD1, RvD2 was described as a potent inhibitor of both TRPV1 and TRPA1 in DRG cells, with a 20-fold higher potency for TRPV1 (IC_50_: 0.1 and 2.1 nM for TRPV1 and TRPA1, respectively). Peripheral and central administration of RvD2 in very low doses (0.01–10 ng) attenuated Complete Freund’s adjuvant-induced inflammatory pain-related spinal long-term potentiation in mice in a TRPV1/TRPA1-independent manner [47]. It has been suggested that pro-resolving mediators such as RvD1 exert activity via specific GPRs, similarly to lipoxin A4 and Resolvin E1 [42]. The RvD1 receptor named DRV1/GPR32 was identified via library screening and with ^3^H-labeled ligand binding [49]. RvD1 was reported as an endogenous inhibitor for the TRPA1, TRPV3, and TRPV4 channels by Ca^2+^ imaging experiments on transfected HEK293T cells and whole cell patch-clamp and Ca^2+^ imaging experiments on mouse DRG sensory neurons. RvD1 suppressed the three TRP channels-mediated nociceptive behaviors in mice [43]. The naturally occurring pro-resolving lipid 17R-RvD1 is a specific inhibitor of TRPV3. The results of Ca^2+^ imaging and whole cell electrophysiology experiments on receptor-expressing HEK cells, sensory neurons, keratinocytes, and behavioral studies suggest that 17R-RvD1 has an acute analgesic effect on TRPV3 ion channels [54]. Intrathecal RvD1 administration prevented postoperative hyperalgesia and mechanical allodynia in a skin–muscle retraction rat model [45]. Intrathecal RvD1 treatment prevented mechanical allodynia in a chronic pancreatitis model [55]. Feng and co-workers suggest that RvD1 attenuated the phosphorylation of NMDA receptor subunits and down-regulated the expression of inflammatory cytokines in the spinal dorsal horn [55]. Pretreatment with the selective Gαi-coupled GPR inhibitor pertussis toxin as well as the G-protein blocker guanosine-5′-(β-thio)-diphosphate sodium salt prevented the RvD2-induced inhibition of TRPV1- and TRPA1-mediated responses on DRG sensory neurons, providing evidence for GPR-mediated actions [47]. Later, GPR18 was identified as the receptor for RvD2 by ^3^H-labeled ligand binding and receptor knockout mice. GPR18 expression was describe on human leukocytes, including polymorphonuclear neutrophil cells, monocytes, and macrophages [48].

On TRPV1-expressing CHO cells, RvD1 and RvD2 induced significant decrease in CAPS-evoked ^45^Ca-accumulation only at 10 nM concentrations. However, both 5 and 10 nM RvD1 and RvD2 significantly and concentration dependently decreased the AITC-induced ^45^Ca-uptake on TRPA1-expressing CHO cells. Since these cells are unlikely to express the resolvin receptors, RvD1 and RvD2 are suggested to inhibit TRP channel activation through surrounding lipid raft disruption. The results that PTX pretreatment did not affect the inhibitory effect of the higher 10 nM doses of RvD1 and RvD2 on TRPV1 and TRPA1 ion channel activation in cultured TG neurons also suggest G-protein-independent way of inhibition. KCl-induced Ca^2+^-influx in TG cells and CGRP release from sensory nerve endings remained unaltered after RvD1 or RvD2 treatment. Our previous results confirmed that lipid raft disruption did not alter KCl-evoked voltage-gated Ca^2+^ channel activation [31]. Here, we provide the first direct evidence by fluorescence spectroscopy for the ability of RvD1 and RvD2 to induce transition from liquid-ordered to liquid-disordered phase in the plasma membrane, indicating cholesterol depletion [30,31,32] and leading to lipid rafts disintegration. High intensity change, but no anisotropy, was observed after both resolvin treatments, indicating less non-radiative processes of Laurdan, less interactions with its micro-environment, and rotational motion restricted to the non-treated control level within the membrane [31,32].

We showed here a dual inhibitory effect of RvD1 and RvD2 on TRPV1 and TRPA1 ion channels via surrounding lipid raft decomposition in the membrane. These results suggest that targeting lipid raft integrity can open novel peripheral analgesic opportunities by decreasing the activation potentials of nociceptors.

## 4. Materials and Methods 

### 4.1. Primary Cultures of TG Neurons

Cells of TG of 1–4 days old Wistar rat pups (6 pups/experiment) were cultured. The ganglia were cut and placed into ice cold phosphate-buffered saline (PBS), incubated for 35 min at 37 °C in PBS containing collagenase (Type XI, 1 mg/mL), and then, in PBS with deoxyribonuclease I (1000 units/mL) for 8 min. The ganglia were then dissociated by trituration. Cell cultures were plated on poly-D-lysin-coated glass coverslips and grown in a nutrient-supplemented medium that contained 180 mL Dulbecco’s-Modified Eagle Medium (DMEM), 20 mL horse serum, 20 mL bovine albumin, 2 mL insulin-transferrin-selenium-S, 3.2 mL putrescin dihydrochloride (100 μg/mL), 20 μL triiodo-thyronine (0.2 mg/mL), 1.24 mL progesterone (0.5 mg/mL), 100 μL penicillin, and 100 μL streptomycin. Cultures were maintained at 37 °C in a humidified atmosphere with 5% CO_2_, and nerve growth factor (NGF, 200 ng/mL) was added every second day, as described earlier [56].

### 4.2. Ratiometric Technique of Intracellular Free Calcium Concentration ([Ca^2+^]_i_) Measurement with the Fluorescent Indicator Fura-2-Acetoxymethyl Ester (Fura-2-AM)

One to three-days-old cell cultures were stained for 30 min at 37 °C with 1 μM of fluorescent Ca^2+^ indicator dye—fura-2-AM (Molecular Probes)—then, washed in extracellular solution (ECS). ECS was gravity fed to the cells using a triple outlet tube and test solutions arrived at the outlet via separate tubes. We could control the rapid changing of solutions by a fast step perfusion system (VC-77SP, Warner Instrument Corporation, Harvard Apparatus GmbH, Freiburg, Germany). Calcium transients of TG neurons to CAPS (stimulation was 10 s) and AITC (stimulation was 15 s) were examined with microfluorimetry, as described elsewhere [56]. Fluorescence images were taken with an Olympus LUMPLAN FI/x20 0.5 W water immersion objective and a digital camera (CCD, SensiCam PCO, Kelheim, Germany), connected to a computer. Cells were illuminated alternately with 340 and 380 nm light generated by a monochromator (Polychrome II., Till Photonics, Kaufbeuren, Germany) under the control of Axon Imaging Workbench 2.1 (AIW, Axon Instruments, CA). We measured the emitted light > 510 nm. The $R\;=\;\frac{F340}{F380}$ was monitored (rate 1 Hz) continuously for up to 2 min, while a few sample images were also recorded. The *R* values were generated by AIW 2.1 software, then, processed by the Origin software version 8.0 (Originlab Corp., Northampton, MA, USA). Baseline was adjusted to R=0, and the peak magnitude of ratiometric responses was calculated. 

Neurons were incubated with 1 μg/mL PTX overnight, 2 and 10 nM RvD1 and RvD2 for 60 min, respectively, at 37 °C in a humidified atmosphere with 5% CO_2_, or were untreated controls. To investigate the voltage-gated ion channel alteration, 50 mM KCl was applied after RvD1 and RvD2 incubation.

### 4.3. Measurement of TRPV1/TRPA1 Activation-Induced CGRP Release from Sensory Nerve Endings of the Isolated Rat Trachea

This method has been described in detail elsewhere [52,53]. In brief, rats (age of 30–40 weeks; in total, 196 animals) were exsanguinated in deep anesthesia (sodium thiobarbital 50 mg/kg intraperitoneal), then, tracheae were removed, cleaned of fat and adhering connective tissues, and placed into an organ bath to achieve sufficient amounts of peptide release and perfused (1 mL/min) with pH 7.2 controlled oxygenated Krebs solution for 60 min (equilibration period) at 37 °C. Then, they were incubated in the presence of RvD1 (10, 50, and 100 nM) or RvD2 (1, 5, and 10 nM) for 60 min or left untreated for control. The trachea is an excellent model to investigate the activation of peripheral nerve terminals. These nerve endings are close to the surface and they can be easily stimulated by different agonists. After discontinuation of the flow, the solution was changed three times for 8 min to produce pre-stimulated, stimulated and post-stimulated fractions. Chemical activation was performed in the second 8 min period, with the selective TRPV1 agonist CAPS (100 nM) or the TRPA1 agonist AITC (100 μM) or KCl (50 mM) to elicit CGRP release. CGRP concentrations were determined from 200 µL samples of organ fluid of the preparations by means of radioimmunoassay methods developed in our laboratories as described [52,53]. CGRP concentrations were measured also from the media of non-stimulated samples in the presence of resolvins to determine their effect on the baseline peptide release. The trachea samples were weighed and CGRP release was calculated as fmol/mg wet tissue. The absolute peptide outflow in response to the chemical stimulations was calculated by adding CGRP release measured in the stimulated and post-stimulated fractions after subtracting the basal release measured in the pre-stimulated 8 min fraction. In each group, 6 experiments were performed per group (12 tracheae per group; 2 tracheae in each organ bath chamber).

### 4.4. Radioactive ^45^Ca-Uptake Experiments in CHO Cells Expressing Cloned TRPV1 or TRPA1 Receptors

CHO cells stably expressing the human TRPV1 or rat TRPA1 receptor were plated into the medium onto Microwell Minitrays (Sigma Inc., Marlborough, MA, USA) in 15 μL cell culture, similarly as described earlier for HT1080 cells [57]. The following day, the cells were washed 5 times with calcium-free Hank’s solution (pH 7.4), then, incubated in 15 μL of the same buffer containing the desired amount of RvD1 and RvD2 (1, 5, and 10 nM) on 37 °C for 60 min. After washing with Hank’s solution, the cells were incubated in 10 μL of the same buffer containing 100 nM CAPS or 100 µM AITC and 200 μCi/mL ^45^Ca isotope (1.3 Ci/mM, Amersham) for 2 min at room temperature. After washing 5 times with ECS, the residual buffer was evaporated. Then, the retained isotope was collected in 15 μL 0.1% sodium dodecyl sulphate and the radioactivity was measured in 2 mL scintillation liquid in a Packard Tri-Carb 2800 TR scintillation counter.

### 4.5. Fluorescence Spectroscopy to Determine Membrane Polarity Related to Lipid Raft Integrity

CHO cells were incubated with Laurdan in 40 µM final concentration for 40 min at 37 °C in a humidified atmosphere with 5% CO_2_. Laurdan is a membrane probe that is sensitive to the polarity of the membrane [40,58]. RvD1 and RvD2 in 100 nM were diluted with ECS and added to the cell wells for 45 min at 37 °C before Laurdan administration, respectively. Cells were then washed 3 times with PBS and scraped from the plates into 1 mL PBS. Fluorescence excitation and emission spectra, excitation–emission matrices, and anisotropy spectra were measured by a HORIBA Jobin-Yvon Nanolog FL3-2iHR spectrofluorometer equipped with a 450 W xenon lamp. Samples were measured in a 4 mm path length quartz cuvette (Hellma 104F-QS) and kept at a constant 20 °C using a Thermo Scientific circulating bath AC200-A25. Excitation–emission matrices consisting of a series of emission spectra recorded at different excitation wavelengths were measured to determine spectral changes. An excitation–emission matrix has one axis for the emission wavelengths, while the other includes the excitation wavelengths. At the intersection points, fluorescence intensity can be read as the value of the third axis. Steady-state emission anisotropy was measured in “L-format” arrangements to study the molecular mobility. Excitation was vertically polarized, while anisotropy was calculated from consecutively measured vertical and horizontally polarized emission intensities. Anisotropy <*r*> is defined as

$$<r>=\frac{I_{VV}{-G*I}_{VH}}{I_{VV}{+2*G*I}_{VH}}$$

where *G* is the spectrofluorometer’s sensitivity factor given by:

$$G=\frac{I_{HV}}{I_{HH}}$$

where *I_HV_* and *I_HH_* are measured using horizontally polarized excitation and vertically and horizontally polarized emission, respectively. The *G* value was automatically recalculated at each wavelength point of the anisotropy measurements.

### 4.6. Drugs and Chemicals

CAPS and AITC (Sigma, St. Louis, MO, USA) were dissolved in dimethyl sulfoxide (DMSO) to obtain a 10 mM stock solution. Further dilutions were made with ECS or Krebs solution to reach final concentrations of 330 and 100 nM in the case of CAPS and 200 or 100 μM in the case of AITC, respectively. RvD1 an RvD2 were purchased from Cayman Chemical in an ethanolic solution. Laurdan was purchased from Sigma and dissolved in (DMSO) to obtain a 10 mM stock solution. PTX was purchased from Sigma and dissolved in ECS. DMEM, horse serum, fetal bovine albumin, and newborn calf serum were purchased from Gibco (Grand Island, NY, USA). Collagenase, deoxyribonuclease I, poly-D-lysine, and NGF were purchased from Sigma.

### 4.7. Statistical Analysis

The Kolmogorov–Smirnov normality test showed normal distribution for all data sets; we performed the analysis of variance (ANOVA) for statistical analysis. Data reported in this paper are the means ± SEM of six to eight independent experiments in the case of Ca^2+^-influx measurement in cultured TG neurons and measurement of CGRP release, and nine independent experiments in the case of ^45^Ca-uptake experiments in CHO cells. In the CGRP release measurements in each group six experiments were performed per group (12 tracheae per group; 2 tracheae in each organ bath chamber). Fluorescence spectroscopy measurements were performed with four samples per group. Statistical analysis was performed by one-way ANOVA with Dunnett’s post hoc test; in all cases *p* < 0.05 was considered statistically significant.

## Abbreviations

[Ca^2+^]_i_	intracellular free calcium concentration
AITC	allyl isothiocyanate
CAPS	capsaicin
CHO	Chinese hamster ovary
CGRP	calcitonin gene-related peptide
DMEM	Dulbecco’s Modified Eagle Medium
DMSO	dimethyl sulfoxide
DRG	dorsal root ganglion
ECS	extracellular solution
fura-2-AM	fura-2-acetoxymethyl ester
GPR	G protein-coupled receptor
Laurdan	6-dodecanoyl-N,N-dimethyl-naphthylamine
NGF	nerve growth factor
PBS	phosphate-buffered saline
PTX	Pertussis toxin
RvD1	Resolvin D1
RvD2	Resolvin D2
SM	sphingomyelin
TG	trigeminal ganglion
TRP	Transient Receptor Potential
TRPA1	Transient Receptor Potential Ankyrin 1
TRPV1	Transient Receptor Potential Vanilloid 1
